# Psychiatric genomics, mental health equity, and intersectionality: A framework for research and practice

**DOI:** 10.3389/fpsyt.2022.1061705

**Published:** 2022-12-21

**Authors:** Julia E. H. Brown, Jennifer L. Young, Nicole Martinez-Martin

**Affiliations:** ^1^School of Nursing, University of California, San Francisco, San Francisco, CA, United States; ^2^Feinberg School of Medicine, Northwestern University, Chicago, IL, United States; ^3^School of Medicine, Stanford University, Stanford, CA, United States

**Keywords:** psychiatric genomics, intersectionality, psychiatric research, genetic counseling, psychiatric treatment, inequity and social justice, structural violence, polygenic risk scores

## Abstract

The causal mechanisms and manifestations of psychiatric illness cannot be neatly narrowed down or quantified for diagnosis and treatment. Large-scale genome-wide association studies (GWAS) might renew hope for locating genetic predictors and producing precision medicines, however such hopes can also distract from appreciating social factors and structural injustices that demand more socially inclusive and equitable approaches to mental healthcare. A more comprehensive approach begins with recognizing that there is no one type of contributor to mental illness and its duration that should be prioritized over another. We argue that, if the search for biological specificity is to complement the need to alleviate the social distress that produces mental health inequities, psychiatric genomics must incorporate an intersectional dimension to models of mental illness across research priorities, scientific frameworks, and clinical applications. We outline an intersectional framework that will guide all professionals working in the expanding field of psychiatric genomics to better incorporate issues of social context, racial and cultural diversity, and downstream ethical considerations into their work.

## Introduction

The causal mechanisms and manifestations of psychiatric illness cannot be neatly narrowed down or quantified for diagnosis and treatment. The field of psychiatric genomics nonetheless aspires to locate “the fundamental basis” of mental illness by using global datasets to identify causal genetic variants and map gene expression in the brain, which can then deliver “actionable knowledge” (1). Multiple copy number variants (CNVs) are increasingly associated with neurodevelopmental conditions such as autism (2), as well as severe mental illness such as schizophrenia (3). Yet all neurodevelopmental and psychiatric disorders appear to have overlapping genetic etiologies (4) and overlapping phenotypes (5). The field of psychiatric genomics has not lead to any substantial improvements in mental healthcare nor the building of comprehensive, ethical, or cost-effective clinical approaches (6, 7). While large-scale genome-wide association studies (GWAS) renew hope for locating genetic predictors and producing precision medicines to improve prevention and long-term outcomes, such aspirations can also distract from societal factors that demand more socially inclusive and equitable approaches to mental healthcare.

Mental health is multi-factorial and disorders are defined in various ways by diagnostic conditions (with clinical knowledge and interpretations changing over time), structural conditions (systemic biases and access to support services), the person diagnosed (who may embrace or reject the label as part of their social identity), and that person’s social ties. Seeing a condition as biological can alleviate social and individual responsibility for its emergence. For instance, autism spectrum disorder can be reclaimed as natural neurodiversity, wherein individuals diagnosed are seen as deserving of family advocacy, social acceptance and structural supports (8). In contrast, the label of schizophrenia has not been widely embraced as a condition of neurodiversity and is instead associated with structural disadvantage and imbued with ideas of social deficit, reinforcing an individual’s disempowerment that precedes and follows diagnosis (9). There is no one type of contributor to mental illness that should be prioritized over another; there is a need to expand conceptual models of mental distress to encompass plural, rather than binary, explanations (10).

If genetic and genomic models prioritize biological processes, this reinforces conceptual binaries and epistemological biases. The “postgenomic” era of science, striving to include environmental, social and behavioral influences, together with epigenetic models that incorporate gene-environment dynamics, provoke questions of “epistemic environments”: How knowledge discourse concerning environmental influences may still be reductive and molecular-oriented to the exclusion of such influences as colonial legacies and racism (11). In psychiatric research, there are growing calls to incorporate social dynamics to improve the likelihood of progress (12, 13), with the COVID-19 pandemic spotlighting the importance of historical and structural determinants of mental health (14).

Beyond genetic associations, a person’s relative social position, social connections and cultural environment impact the etiology, expression, and progression of mental illness, with structural racism playing a significant role (15). To take the example of schizophrenia, there are at least 145 associated CNVs (16), none of which are specific to the condition (17). On average, there are 100 published studies on the genetics of schizophrenia each year, with expanding genetic databases and GWAS dedicated to comprehension and improving the clinical relevance of such studies (18). When using datasets that are inclusive of African as well as European and Latinx ancestry, polygenic risk prediction for schizophrenia, like for other conditions, is substantially weakened for individuals classified as having African ancestry compared to those identified as European or Latinx (19). Biochemical links to schizophrenia such as prenatal vitamin D deficiency may or may not be resolved through larger population representative sets such as using an entire population’s newborn genetic screening data (20), because such specific biochemical associations are embedded in larger webs of social influences on health behaviors. Improving genetic ancestry representation would not improve comprehension of how social and cultural differences and marginalization shape the course of illness. The prevalence of schizophrenia varies across social contexts and correlates with circumstances of birth, experiences of trauma and social adversity, migration, urban settings and substance abuse (17, 21). There may be a “patho-biography” at play, where social traumas shape neurodevelopment (22). In terms of treatment, opportunities for social empowerment amongst individuals diagnosed with schizophrenia may play more of a key role in recovery than biomedical interventions alone (9, 23–25). Theories regarding genomics and mental health therefore need to be framed in ways that better address how biological factors *intersect* with culture and environment, including systems of social opression (26).

**FIGURE 1:**
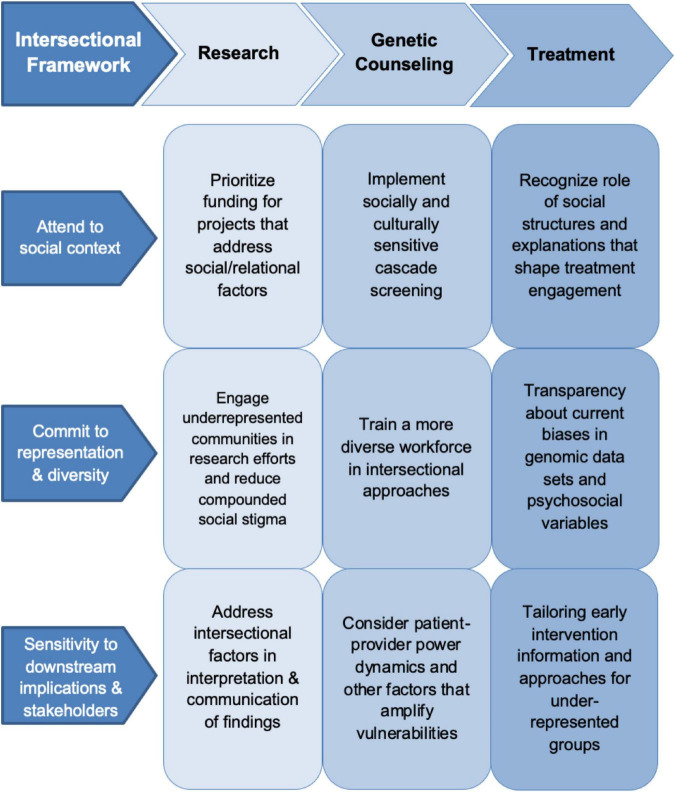
Intersectional framework psychiatric genomics.

Here we argue that if the search for biological specificity, using knowledge frameworks that prioritize molecular-level environmental influences over social power dynamics, is to complement the need to alleviate the social distress that produces mental health inequities, psychiatric genomics must incorporate an *intersectionality* dimension to models of mental illness across research priorities, scientific frameworks, and clinical applications. Intersectionality captures how multiple categories of social identity—such as racialized minority, class, gender, age, disability status, family position or social connections—shape a person’s experience and treatment in society (27). In a clinical context, this means attending to the ways in which patients, as people-in-context, can be marginalized through systemic, discriminatory practices (28). An intersectional analysis can facilitate research across relevant health categories and allows for an examination of how interacting social dimensions influence mental health (29). An intersectional approach can illuminate how intersecting socially constructed categories, such as race, class, or gender, impact mental health at the individual and population level (30, 31). Below we detail how an intersectional framework can support psychiatric genomics across three key stages of psychiatric genomic research and knowledge translation: (1) Genomic research practices; (2) genetic counseling for patients and families; and (3) in enhancing biomedical models of psychiatric care (Figure 1).

### Adding intersectionality to genomic research practices

The first task will be bringing together useful interdisciplinary concepts that capture why intersectional factors are relevant to mental health. Just as the field of genomic medicine more generally will not improve health equity until a common language about health disparity is established between researchers (32), psychiatric research cannot improve without finding such language. The intersectional aspects of mental distress cannot, by definition, be narrowed down to any one factor; the idea is not to pinpoint factors but rather to contextualize. There are no clear answers to explain why Black Caribbean immigrants in London are 15 times more likely than white Londoners to be diagnosed with schizophrenia (33), or why those in non-industrial societies may have a 50 per cent higher chance of recovery from schizophrenia than those in industrial societies (24). These findings are not simply about racism, genetics, culture and/or the politics of diagnosis and recovery, although each can play a role. A person’s context—their social position, connections and cultural perspective—are influenced by the multiple categories they occupy, such as being a Black Caribbean female immigrant living in London in supported housing and navigating an unreliable mental healthcare system and socially unforgiving diagnostic label of schizophrenia.

In terms of framing how intersectional factors coalesce to produce mental illness, there are at least two interdisciplinary concepts that can improve communication about complex biopsychosocial interactions. The first is structural violence, which refers to how conditions inflicted by unequal social structures and institutions fail to look after basic human needs, resulting in illness (34), including severe mental illness like schizophrenia (35). The second way of capturing the embodied impact of social disparity on mental health is through the lived experience of social defeat. Social defeat refers to how the feeling of being an “outsider” or a “failure” in social settings often precedes psychosis, addiction, depression, and psychosis which may explain in part why psychosis is more common amongst migrant communities and people with less opportunity to experience social cohesion (23, 36). Further, in the absence of phenotypes and clear markers of genetic vulnerability or social vulnerability, models of social defeat may help to improve upon epigenetic models that explore how intergenerational stressors alter DNA but ultimately redefine environmental factors in terms of molecular biology (37, 38).

Establishing the usefulness of interdisciplinary concepts cannot happen without expanding what types of research receive federal funding and who is funded to do this research. In general, the U.S. National Institute of Health has been less likely to award grants to research proposals that are aimed at the subjective experiences of oppressed communities or populations (39). This gap has contributed to lower rates of diversity among researchers. There is also a lack of representation of researchers with lived experience: The National Institute of Mental Health has called for the expansion of research workforce diversity without including researchers with lived experience in that call (40). In terms of research design, efforts must also be made to directly involve non-academic community members with lived experience. For example, involving people with lived experiences in putting together Digital Storytelling narratives that can be used to educate others about what mental health conditions mean to people diagnosed (41).

Building in an intersectional approach means improving both diversity of research participants and accounting for biases in data collection. The current lack of diversity in the research participant population can lead to reduced downstream benefit for marginalized communities (42). In addition to the inability to generalize GWAS findings and applications due to disparities in ancestry representation in biobanks, there are inherent biases within electronic health record data such as the subjective diagnoses of phenotypic traits, which then “infer differences between groups that are a result of structured bias rather than biological truth” (43). Intersectional approaches to research questions can provide a process for questioning how diagnostic information was gathered, the social demographics to whom it was applied, and when it is appropriate to analyze according to racialized categories (44), as well as take into account additional relevant social categories that give a better understanding of the structural and relational factors that influence psychiatric illness.

Researchers—whether with lived experiences or not—should actively reflect on how social and structural factors potentially contribute to the subject of study. For example, a recent GWAS examining the genetic underpinnings of depression pooled together genetic information from over 1.2 million research participants to find a total of 178 “genetic risk loci,” results of which were affirmed by running a subsequent biobank analysis and discovering potential overlap with pharmacological targets (45). Arguably, this large number of genetic loci might equally suggest how common these genetic loci may be amongst the general population and implicate many complex social interactions. Incorporating shared social as well as genetic variables into analyses can account for intersectional and relational factors (30). While quantifying social data may not be appropriate or possible for some research questions, intersectional issues can still be engaged in other stages of the research process, including the interpretation and communication of research findings.

More engagement with the concerns of affected communities regarding the goals, methods and outcomes of psychiatric genetic research is needed, to recognize the humanity in people behind the data points. For example, there have long been questions regarding higher rates of diagnosis of schizophrenia in Black and Latinx men—whether this reflects overdiagnosis or greater psycho-social stressors on those groups (46, 47). Gender norms, and particularly masculinity, can intersect with structural factors to produce mental illness (48). It is paramount that the reporting of such findings aims toward alleviating psychiatric stigma and biological essentialism, to instead emphasize social complexities. Preserving the dignity of the people whose situations pertain to such research findings requires active efforts to humanize rather than stigmatize, and to not merely let the data tell the story but rather bring in the voices behind the data where possible.

### Intersectionality in counseling for patients and families

Outside of clinical psychiatry, genetic counselors increasingly interface with patients receiving genetic diagnoses, especially with regards to gathering family history data, supporting decision making, and returning test results. As genomic testing and technology, such as polygenic risk scores, expands to psychiatry, there will be an onus on genetic counselors to explain environmental as well as genetic factors that contribute to illness risks and subsequent management of these risks (49). Although there has been an increased demand for psychiatric genetic counseling, genetic counselors rarely receive primary referrals for this service and report feeling discomfort providing genetic counseling for psychiatric illness (50, 51).

In comprehending and communicating the breadth of risk, genetic counselors need to be better educated on interpersonal power dynamics—such as those that produce social defeat and structural violence—and on how their own relationships exist with systems of inequality. The field of genetic counseling itself lacks diversity, with 90 percent of genetic counselors from a 2021 survey identifying as non-Hispanic White (52). Genetic counseling training programs have recognized the importance of a socially inclusive workforce, but until that is achieved, intersectional methodologies for addressing patient-provider power hierarchies will be limited.

As the patient-facing provider in psychiatric genetics, genetic counselors must be prepared to assess and address complex family dynamics, especially for patients of different backgrounds who lack traditional support systems. People with severe mental health conditions may be completely cut off from family members or else highly dependent on family for caregiving, financial assistance, and psychological/emotional support, and some may even have lost their legal ability to function independent of family. Family values and behaviors are often sanctioned by peoples’ ethnic background and thus family conflict may be related to values that have not been upheld by the person with the mental health condition (53). For example, for their distress Latinx families may be less likely to blame an ill family member but may be more sensitive to behaviors such as alcohol or drug use that disrupt harmony in the home and can cause interpersonal aggressiveness (54). High expressed emotion in families (represented by hostility, criticism, and emotional over-involvement) is the third most common cause of relapse in schizophrenia, after adherence with medication and drug abuse (55). While high expressed emotion has negative connotations in this context, research has shown that Black families actually might experience critical and intrusive behavior as a symbol of engagement, caring, and support (56). Moreover, emotional warmth and more time spent with family can be protective against psychosis relapse for Mexican American families but not Euro-American families (57). These ethnic and racial differences come down to social and cultural factors rather the biological factors like genetic ancestry.

Families play a crucial but complicated role in management of mental health illness. One of the strongest motivations cited for undergoing genetic testing is a sense of familial obligation, the possibility of benefit for other family members (58). Since it is likely that multiple family members have been affected by the mental health condition, either *via* someone with a diagnosis or having experienced symptoms themselves, the decision to undergo testing may be considered as a family matter from the outset, including an assessment of the educational and support needs not only of the proband, but of all family members. Each family member’s genetic risk information can have significant implications for other members of the family. Historically, family and twin studies have demonstrated that some major psychiatric disorders aggregate in families and are likely heritable (59). In the current genetics landscape, multiple family members may engage in the genetic counseling and genetic testing process. This is a process called cascade genetic testing, which systematically identifies and tests at-risk family members, but often relies on family communication of risk. Cascade genetic testing is a cost-effective preventative health intervention, but due to the reliance on family communication does not have equitable levels of uptake in some communities of color, with Asian and Black American family members engaging in cascade genetic testing at lower rates (60–62). Only with the support of family and communities can genetic testing have broader reach and more equitable distribution of benefits.

### Enhancing biomedical psychiatric treatment approaches

As well as genetic counseling to inform patients and families of potential risks and management strategies, psychiatric treatment interventions spanning early intervention to chronic illness need to engage multi-factorial influences on treatment outcomes. Emergent genetic technologies are anticipated to become central to psychiatric care—from routine testing of rare genetic variants and polygenic risk profiles, to screening for a likely therapeutic response or side effects attached to psychotropic medication (63). Currently, it is primarily social factors that are revealed in psychiatric consultations that accumulate a patient’s history. As genetic information becomes more available to inform patient history, it is critical that social factors (socioeconomic position, race, gender, and family support) become equally central to prevention and treatment approaches. This is especially so given that predictive technologies like polygenic risk scores reveal only one of many determinants, with accuracy stronger for European ancestry groups, with determinants inevitably made more or less significant depending on life events and experience (64). While precision psychiatry if accurate enough has the potential to save lives, clinical application of novel approaches must be understood as incomplete “companion decision-support tool[s]” that cannot simply compensate for non-genetic approaches (65). Treatment biases that privilege biological explanations and/or some population groups over others and issues of genetic ancestry representation and applicability must be continually checked and challenged.

When it comes to medications, targeting a psychiatric condition through more refined medications or precision medicine does not confer a stable relationship between a biological treatment target and the prescribed drug because medications become part of a social ecosystem. Whether it is protocol or not, clinicians and patients work with explanations that make sense in the immediate context. For example, in Brazil, doctors began prescribing SSRIs not for depression or biochemical instability but rather “for the suffering caused by the social situation,” understood to be imposed by economic and political upheaval (66). Amongst socially disadvantaged youth diagnosed with mental illness in New Mexico, the social reasoning for taking prescribed psychotropic medication revolves around an “engaged struggle” to manage emotion and behavior, where social adversity and trauma that is nonetheless understood to have contributed to the initial diagnosis is left unaccounted except for through family management (67). Because increased genetic emphasis on diagnostics and treatments does not, in practice, simply incline clinicians or patients toward biological reductionism (66), practitioners of new technologies such as pharmacogenomics (the tailoring of psychopharmaceuticals to match a patient’s genomic profile in order to improve efficacy and reduce side effects) might benefit from anticipating social and structural influences as part of predictive modeling.

It is also important to keep humanizing mental health conditions in the process of treating them. At the extreme, when a clinician views a condition such as schizophrenia as primarily biological or genetic, they are likely to fail to empathize with the patient and this in turn de-humanizes them (68). One way in which patients can be re-humanized in the course of biologically focused treatments, and particularly treatments for chronic psychiatric illness, is by shifting the focus toward other more relatable aspects of ill health. For example, in the United Kingdom and Australia, clozapine treatment for psychosis involves regular visits to clozapine clinics for physiological monitoring. This routine monitoring inadvertently offers what a clozapine client described as “routine connection” to other people, paving the way for structural support and social empowerment that can improve wellbeing as much as the biochemical help from clozapine (69). In the course of emergent genetic technologies becoming direct-to-consumer, we cannot afford to lose these clinical environments that provide social opportunities for patients.

Finally, the delivery of prognostic information from machine learning tools must include psycho-social variables and biases. There is a psychological effect of disclosing risk information to patients, who may interpret information differently, and clinicians must decipher whether providing the information conforms with the ethical principles of respect for autonomy and beneficence (70). First, individual and group differences must be taken into account when using any genomic markers to guide medication dosing (71). For example, “early intervention” approaches to psychosis through “at-risk clinics” do not include enough African Americans and Asian Americans for any evidence of positive treatment responses to be accurate and representative for these population groups (72).

Second, respecting patients and working with the intersectional social factors that are specific to them (gender, race, culture, etc.) means being honest about scientific biases and inviting conversation about alternative belief systems. For example, in the case of genetic predictors for suicide, the question of prevention can never be reduced to first locating a phenotype, of which there is none, and preventive “treatments” will ultimately depend on social receptiveness to that intervention (73). As molecular testing is incorporated into treatment protocols, particularly regarding personalized medicine wherein doses of medication and estimation of risks can be inferred through genomic tests, it is critical that psychiatrists promote shared decision-making models. This means allowing for patient values to become a central part of decisions (74).

## Conclusion

This article has argued for an intersectional dimension to be added to psychiatric genomics across the domains of research, genetic counseling and precision-oriented treatments. Currently, emergent psychiatric genetic technologies focus too much attention on biological causes and interventions for mental disorders, without also addressing the many cultural and interpersonal factors that contribute to mental wellbeing. There are significant social and racial disparities in mental health and psychiatric genomic medicine must actively work to alleviate rather than exacerbate these disparities. The complex genetic contributors, including polygenic risk correlations, drug metabolism and ancestry, must be seen in the context of intersecting structural factors, including social position, race/ethnicity, family relations, cultural needs, and healthcare access. The intersectional framework we propose has implications for all professionals working in the field of psychiatric genomics, who must incorporate issues of social context, diversity, and downstream implications into their work. Incorporating the intersectional dynamics of biological and relational/structural stressors into research and translation will help to ensure that advancements in psychiatric genomics are more inclusive for currently marginalized populations. A failure to do so will be highly consequential for society, and especially those living with or at risk of serious mental illness, and often chronic co-morbidities, who face multiple systemic barriers to equitable healthcare and social support.

## Data availability statement

The original contributions presented in this study are included in the article/supplementary material, further inquiries can be directed to the corresponding author.

## Author contributions

JB took the lead on the writing. JY and NM-M contributed to each section. NM-M was the lead of this collaboration. All authors contributed to this manuscript and approved the submitted version.
